# Morphological Characteristics of Biopolymer Thin Films Swollen-Rich in Solvent Vapors

**DOI:** 10.3390/biomimetics9070396

**Published:** 2024-06-30

**Authors:** Mihai Băbuțan, Ioan Botiz

**Affiliations:** 1 Department of Physics of Condensed Matter and Advanced Technologies, Faculty of Physics, Babeș-Bolyai University, 400084 Cluj-Napoca, Romania; mihai.babutan@stud.ubbcluj.ro; 2 Interdisciplinary Research Institute on Bio-Nano-Sciences, Babeș-Bolyai University, 400271 Cluj-Napoca, Romania

**Keywords:** biopolymers, thin films, solvent vapor annealing, crystallization, self-assembly, microstructure

## Abstract

Biopolymers exhibit a large variety of attractive properties including biocompatibility, flexibility, gelation ability, and low cost. Therefore, especially in more recent years, they have become highly suitable for a wider and wider range of applications stretching across several key sectors such as those related to food packaging, pharmaceutic, and medical industries, just to name a few. Moreover, biopolymers’ properties are known to be strongly dependent on the molecular arrangements adopted by such chains at the nanoscale and microscale. Fortunately, these arrangements can be altered and eventually optimized through a plethora of more or less efficient polymer processing methods. Here, we used a space-confined solvent vapor annealing (C-SVA) method to subject various biopolymers to rich swelling in solvent vapors in order to favor their further crystallization or self-assembly, with the final aim of obtaining thin biopolymer films exhibiting more ordered chain conformations. The results obtained by atomic force microscopy revealed that while the gelatin biopolymer nucleated and then crystallized into granular compact structures, other biopolymers preferred to self-assemble into (curved) lamellar rows composed of spherical nanoparticles (glycogen and chitosan) or into more complex helix-resembling morphologies (phytagel). The capability of the C-SVA processing method to favor crystallization and to induce self-assembly in various biopolymeric species or even monomeric units further emphasizes its great potential in the future structuring of a variety of biological (macro)molecules.

## 1. Introduction

In recent years, with the awareness of product sustainability problems, biopolymers have started to be used more and more often in the manufacture of different types of biodegradable materials [1,2,3,4]. This was also possible because biopolymers are relatively easy to procure, as they are produced by living organisms in the form of saccharides (such as chitosan, dextrin, glycogen, or phytagel) [5,6,7], proteins (examples here may include gelatin, collagen, or fibroin) [8,9,10,11] or DNA [12,13]. Moreover, because of their strength, steadiness, flexibility, and biocompatibility, biopolymers have a high utility value in the food industry [14,15], in medical applications such as drug delivery [16,17,18,19,20], hydrogel and scaffold production for cell growth and tissue engineering [21,22,23,24], in the construction of various functional biomaterials [19,25], as therapeutic and contrast agents [26,27,28,29], or in other fields of application [30,31], etc. For instance, chitosan is a well-known biopolymer, mostly adopting fibrillar or porous structures when cast on solid surfaces, that is used in drug delivery and anti-microbial applications [32,33]. Dextrin and glycogen, although sharing the same monomer (glucose), can be utilized in a variety of sectors, including food packaging and electronic applications [34,35]. Other notable biopolymers are phytagel, gelatin, and polydopamine. While phytagel is a saccharide that plays multiple roles in the fabrication of hydrogels and scaffolds [20,36,37], gelatin is mostly employed to design and develop medical applications like drug delivery [38,39] and is able to display typical coiled-like conformations such as a nanoparticle configuration when deposited on solid surfaces [40]. Polydopamine is a biopolymer exhibiting novel functions because of its chemical structure comprised of a catechol fraction and an amine side. Considering its functional groups, polydopamine has an important role in the construction of multifunctional hydrogels (including modified hydrogels for medical treatments), where it can be integrated as a stabilizer in the hydrogel network via three different structures including chains, coatings, and nanoparticles [41,42]. Obviously, polydopamine is composed of dopamine monomer units. The latter can assemble into highly ordered structures that can be further used, for example, in combination with other (bio)polymers, in the development of novel composite materials for spinal cord injuries, bioadhesive, and biomimetic and antibacterial applications [43,44,45,46], just to name a few.

What all these biopolymers have in common is the fact that their properties strongly depend on the final (film) microstructure and, thus, on the molecular arrangements adopted by biopolymer chains both at the nanoscale and microscale. Therefore, in order to maximize the use of (bio)polymers in any type of application, it is necessary to exploit the structure–processing–property relationship (and, eventually, to manipulate and clearly understand their ordering into various micro-/nanostructures), as well as the correlation between these resulting structures and their properties at the macroscopic level. Thus, one has to always find, typically for each biopolymer system, an appropriate processing method to generate a specific desired microstructure. Fortunately, nowadays, there is a large variety of more or less efficient processing techniques reported in the literature, with most of them being based inclusively on fundamental physical processes such as self-assembly [47,48] and crystallization [49] (these two processes were recently utilized in the construction of various functional biomaterials and gels exhibiting a high potential in the treatment of wounds and diseases [50,51,52,53]). For example, such (bio)polymer processing methods may rely on thermal and melt-annealing, solvent vapor annealing, hot pressing, or the utilization of space confinements.

Here, we propose to alter and eventually optimize the film microstructure of various biopolymeric systems by employing a processing method based on solvent vapor annealing in a quasi-confined environment (C-SVA) that is capable of favoring and/or inducing the self-assembly and crystallization processes. This method is based on the idea of the rich swelling of (bio)polymer thin films in solvent vapors [54,55], and it was recently further validated on different polymer systems such as various diblock and triblock copolymers [56]. The utilized C-SVA equipment consists of a shallow aluminum sample chamber that is sealed with a glass cover and connected to a source of solvent vapors. The bottom of the chamber is placed on a temperature control mechanism composed of a Peltier element, a state-of-the-art temperature controller, and a temperature sensor so that the temperature of the sample sitting on the bottom of the chamber can be maintained at a specific setpoint within a variation of only 0.01 °C for as long as it is needed. Generally, thin films of a specific biopolymer are inserted into the sample chamber, which is then filled with solvent vapors. Furthermore, by lowering the sample temperature, the solvent vapors are forced to condense onto the film sample. As a consequence, the film undergoes rich swelling until it transforms into a “quasi-two-dimensional (2D) film solution”. Later on, by reversing the current direction in the Peltier element, the sample temperature can increase as slowly as 0.01 °C/s, allowing the solvent molecules to evaporate at a really low rate and thus favoring the films to self-assemble and/or crystallize into (highly) ordered structures.

## 2. Materials and Methods

### 2.1. Description of Biopolymeric Systems Used in This Work

**Figure 1:**
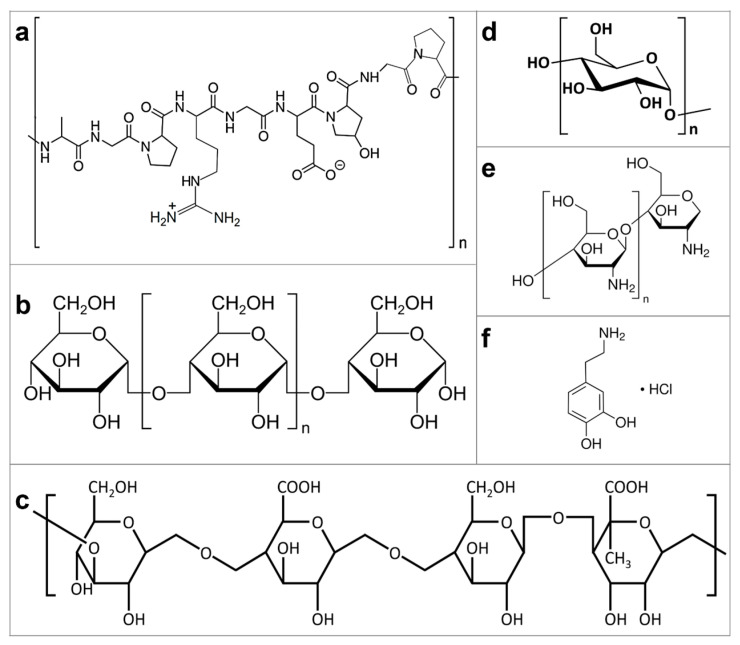
Chemical structures of different biological systems that have been used in this work: gelatin (**a**), dextrin (**b**), phytagel (**c**), glycogen (**d**), chitosan (**e**), and dopamine hydrochloride (**f**).

The biopolymer systems used in this work were purchased from Sigma-Aldrich (Burlington, MA, USA). Dextrin (product number 31400) is a sweet sugar extracted from potato starch and has the linear formula (C_6_H_12_O_6_)_x_. A solution of dextrin was prepared by stirring, at room temperature, 1.5 g of dextrin powder in 30 mL of ultrapure water (solubility 0.5 g in 10 mL of hot water; the white- to faint yellow-colored powder leads to an almost colorless solution at the solubility limit). Glycogen (product number G8751), which, in this case, was extracted from oysters, is a branched polymer of glucose synthesized by animal cells for energy storage and release. A solution of glycogen was obtained by dissolving 6.75 g of this product in 50 mL of ultrapure water. Using the same method, a phytagel (also known as gellan gum, product number P8169; molecular weight 1000 kg/mol; solubility 10 mg in 1 mL of hot water; white- to off-white-colored powder leads to a colorless to faint yellow solution at the solubility limit) solution of a concentration of 10 mg/mL was prepared in ultrapure hot water. Furthermore, solutions of gelatin (product number G9391; solubility 50 mg in 1 mL of water; at the solubility limit, gelatin solution exhibits a faint yellow to yellow or brown-yellow color) and dopamine hydrochloride (product number H8502; molecular weight 189.64 g/mol; solubility 100 mg in 1 mL of water; the white to yellow with a tan cast-colored powder leads to a colorless to dark yellow and colorless to light brown and yellow-brown solution at the solubility limit) were also prepared using ultrapure water-based concentrations of 10 mg/mL and 20 mg/mL, respectively. Finally, water was substituted for acetic acid as a solvent and a 10 mg/mL solution of chitosan of medium molecular weight (with a range of 190–310 kDa, product number 448877) was prepared. In order to favor the complete dissolution of the biopolymers in the chosen solvent, often, the resulting polymeric solutions were additionally subjected to annealing at a temperature of about 70 °C in a silicon oil bath (ONE 7-45, Schwabach, Germany) for 30 min, followed by filtering with 0.22 μm or 0.45 μm polyvinylidene fluoride/polytetrafluoroethylene microfilters from Millipore. While we cannot exclude the presence of some aggregates in such concentrated solutions prior to filtering, after this procedure, all biopolymeric solutions appeared homogeneous but sensitively less concentrated and highly soluble. The chemical structures of all the above biopolymers and dopamine hydrochloride are presented in Figure 1.

### 2.2. Fabrication of Biopolymer Solutions and Thin Films

Each biopolymer solution was dropped onto UV–ozone cleaned silicon wafers (type 4P0/5–10/380 ± 15/SSP/TTV < 5 from Siegert Wafer (Aachen, Germany)) and further spin-cast at 2000 rpm for 30 s using a WS-650mz23nppb spin coater from Laurell Technologies Corporation (North Wales, PA, USA). This procedure led to the generation of homogeneous biopolymeric films that displayed a thickness of about 116 ± 8 nm, 111 ± 7 nm, 93 ± 5 nm, 89 ± 5 nm, 97 ± 6 nm, and 86 ± 5 nm for the cases of glycogen, dextrin, phytagel, gelatin, dopamine, and chitosan, respectively. Each film thickness was determined by measuring the cross-sectional profile of a scratch using the atomic force microscopy (AFM) technique. Note that before filtering the solutions, spin casting led to films of bad quality, with many large and dense aggregates being spread across the whole surface of biopolymeric films. This indicated that water was not an ideal solvent for such biopolymers (neither was the acetic acid for chitosan). After filtering the undissolved biopolymeric aggregates from the solutions, highly homogeneous and uniform thin films were obtained. The main characteristics of both the utilized biopolymer systems and their corresponding spin-cast films are summarized in Table 1.

### 2.3. Processing of Biopolymer Thin Films via C-SVA Method

All films were further subjected to C-SVA processing. Using a homemade experimental setup, that consisted of an aluminum sample chamber with a depth of less than one millimeter and a high-performance Peltier element (15.4 V/8.5 A from Stonecold (Conway, AR, USA)) beneath, thin biopolymer films were swollen-rich in solvent vapors by taking advantage of the condensation phenomenon, followed by a gradual evaporation of all solvent vapors. While this procedure enforced the appearance of either the self-assembly or crystallization process, we do not expect that it induced chemical alterations of our biopolymers (except for the case of dopamine hydrochloride, which is expected to polymerize upon spin-casting and further C-SVA processing into polydopamine). More precisely, the temperature of the probe was regulated by an advanced controller (TCM U 10A from Electron Dynamics Ltd., Southampton, UK), which received feedback from a PT100 temperature sensor located in the (sealed) sample chamber in the vicinity of the sample (more details about the experimental setup can be found in [56]). Once a nitrogen bubbling system finalized the accurate generation and regulation of the amount of solvent vapors in the sample chamber (while the film sample was kept at an elevated temperature of 40 °C; note that the two functions of nitrogen gas are the rapid generation of solvent vapors and their further driving to the sample chamber), the sample temperature was lowered (at a rate of 0.3 °C/s) to 18–22 °C (note here that the exact minimum of the sample temperature usually depended strongly on the type of solvent and its volatility, as well as on the type of processed biopolymers) in order to induce the condensation of solvent vapors onto the film sample. Condensation of solvent vapors is always accompanied by a change in the interference colors (visible only when non-transparent substrates such as silicon wafers are used) that can be observed under an optical microscope or by naked eyes because of the significant increase in the film’s thickness (more details on this phenomenon and procedures to evaluate the thickness of a film upon its swelling and de-swelling can be found in [54]). Once the film was rich-swollen by condensed solvent vapors and became a quasi-2D “film solution”, the film sample was slowly heated (using a heating rate of 0.01 °C/s) back to 40 °C. This procedure triggered a very slow and controlled evaporation of solvent molecules and thus induced and/or favored processes like self-assembly or crystallization. This way, the orderly structuring of the biomolecules was fully accomplished. Once all solvent molecules were completely removed from the biopolymer film, the newly dried film exhibited the same thickness as before its swelling but with a considerably altered and/or optimized film microstructure.

It is important to note that we always started the C-SVA experiments with freshly spin-cast biopolymeric films, with their initial thickness measured by AFM after gently scratching each film. At this point, we considered the polymer concentration (*c_p_*) to be 100%, as it was assumed that no solvent molecules were present in the film. Moreover, each initial film displayed a well-defined color under the optical microscope or naked eyes that corresponded to the initial film thickness. When a certain biopolymer film started to swell upon the condensation of solvent vapors on its surface, the *c_p_* decreased and the color of the film under the optical microscope changed because of the associated interference phenomenon (the change in interference colors is generally visible on non-transparent silicon wafers). Therefore, the approximate *c_p_* in the swollen film could be estimated by observing the color of the swollen film and assessing it to a specific film thickness, previously measured by AFM on “calibrating” films (see all the necessary details on this procedure reported here [54,55]). The *c_p_* was determined as the ratio between the initial and the swollen film thickness. Typically, each biopolymer film was swollen until it reached a thickness of about 2000 nanometers and thus, a *c_p_* of about 5% (at this stage we had a quasi-2D “film solution” with biopolymeric chains being roughly re-dissolved). All biopolymer films were kept at this *c_p_* for about one minute and then the solvent condensation process was reversed by slowly increasing the temperature of the film, followed by the corresponding change in the film color, a decrease in film thickness, and, thus, the slow increase in *c_p_*. It is essential to add here that the structuring of thin biopolymeric films took place when the *c_p_* ranged between about 5% and around 30%. A typical time ranging between less than 10 min and about 16 min was needed to structure each thin biopolymeric film.

All thin biopolymeric films, except for the dextrin films, appeared to be highly soluble and at the same time stable upon the spin-casting and C-SVA processing conditions, thus implying a good adherence/interaction to/with the substrate. Dextrin was the only film presented in this paper that showed signs of dewetting upon C-SVA processing. We also processed other biopolymers, not presented in this work, such as starch, κ-Carrageenan, cellulose, and agar, but the resulting films proved to be unstable even upon spin-casting processing.

### 2.4. Characterization of Thin Films of Biopolymers Using the Optical and AFM Techniques

AFM measurements were conducted using a system from Molecular Devices and Tools for Nano Technology (NT-MDT) fixed on an Olympus IX71 optical microscope operating in non-contact (tapping) mode. The whole AFM system was acquired from Spectrum Instruments Ltd. (Limerick, Ireland). High-resolution Noncontact Golden Silicon probes from NT-MDT, with a tip radius smaller than 10 nm and a tip height ranging between 14 and 16 μm, were used to visualize the biopolymeric surfaces. These probes were coated with gold on the cantilever’s detector side and displayed a length of 125 ± 5 μm and a resonance frequency of 187–230 kHz. They further exhibited a nominal force constant between 1.45 and 15.1 N/m. Moreover, for softer samples, NANOSENSOR PPPNCSTR probes with tip radius smaller than 7 nm were employed, featuring dimensions of 150/27/2.8 μm (length/width/thickness), a force constant of 7.4 N, and a resonance frequency of 75 kHz to 265 kHz. AFM images were captured at a 256 × 256 line configuration, with a scanning speed between 1 and 2 μm/s and a setpoint voltage of 9–12 V, adjusted to maintain a consistently soft tapping regime.

All optical micrographs of biopolymer films were obtained employing a KERN OKN-177 optical microscope (Kern & Sohn GmbH, Balingen, Germany) working in the reflectivity mode and equipped with various magnification objectives, along with a KERN ODC 825 (5 Mp) camera (connected to a computer via Microscope VIS software).

## 3. Result and Discussion

**Figure 2:**
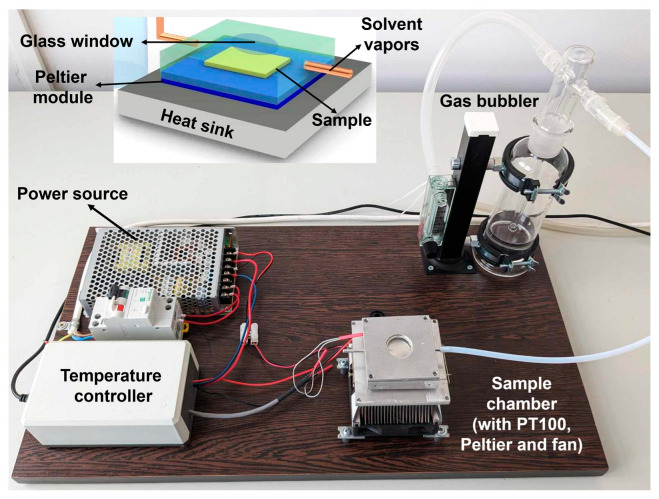
Digital photograph of the homemade C-SVA experimental setup used in this work. The setup comprises an aluminum sample chamber (equipped with a PT100 temperature sensor, a Peltier element, and a heat evacuating fan), a temperature controller, a power source, and a gas (e.g., nitrogen) bubbling system. The latter is capable of introducing a well-controlled amount of solvent vapors into the sample chamber. The inset emphasizes the simplified schematics of the C-SVA chamber.

In order to continuously extend the range of utility of various biopolymers, one has to alter and eventually optimize their (surface) microstructure, along with the corresponding molecular arrangements at the nanoscale and microscale. In this work, we employed a processing method based on the rich swelling of thin biopolymer films in solvent vapors known as C-SVA. The experimental setup utilized in this work is shown in Figure 2 and is already well described in the above section (detailed extra information on the experimental setup, working procedures, and processing efficiency on other polymeric systems can be found elsewhere [55,56]). Briefly, the equipment was composed of a sample chamber equipped with a temperature sensor comprised inclusively of an aluminum bottom of well-controlled temperature, a solvent vapor bubbling system, a temperature controller, and a source of current. With this homemade system, thin films of various biopolymers were rich-swollen through the condensation of warmer solvent vapors onto the colder film surface, allowing for the involved biomolecules to become dispersed and highly mobile. Once the solvent vapors began to be gradually removed by heating the sample very slowly, the biomolecules started to interact more closely and adopt more ordered and/or highly crystalline chain conformations, eventually leading to the formation of various crystals or self-assembled structures.

**Figure 3:**
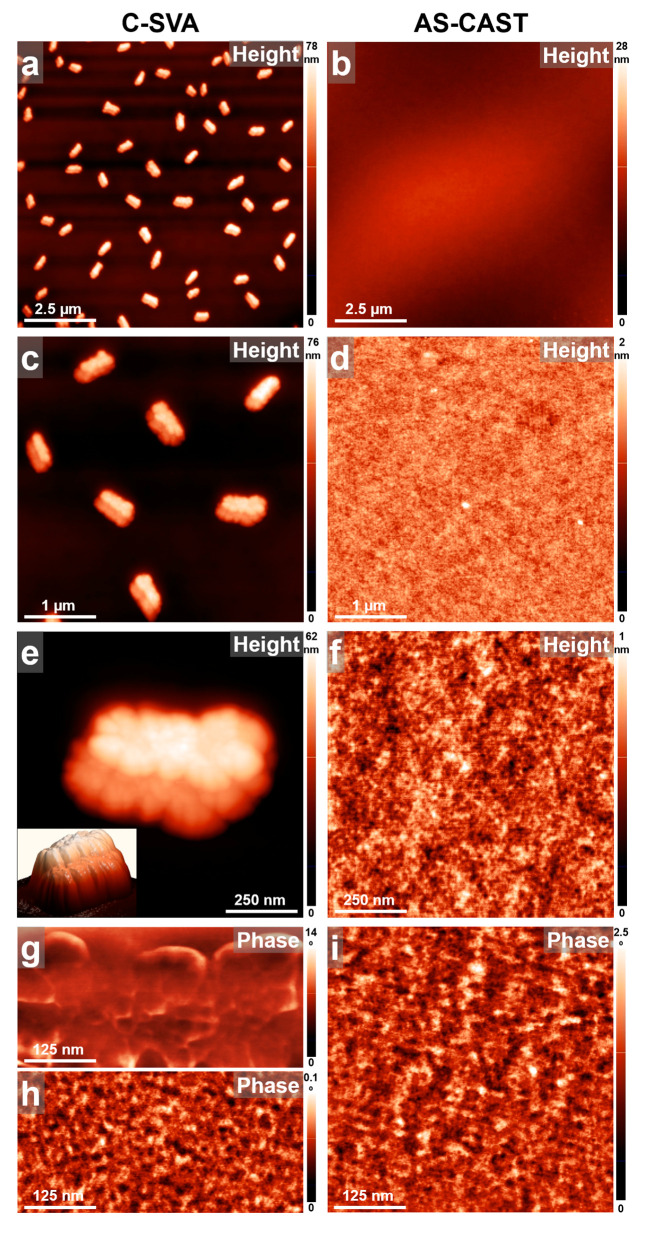
(**a**–**i**) AFM height (**a**–**f**) and phase (**g**–**i**) micrographs illustrating the morphology observed on the surface of a thin film of gelatin before (**right side**) and after (**left side**) being exposed to ultrapure water vapors via C-SVA. After C-SVA exposure, gelatin films display a morphology comprising granular-like structures of elongated shape randomly distributed over the whole surface.

We start our discussion by first presenting the case of gelatin biopolymer. As it can be observed in the AFM images displayed in Figure 3a,c, exposing a thin film of gelatin to ultrapure water vapors via the C-SVA technique led to the formation of granular-like structures of elongated shape. These structures exhibited a length of about 695 ± 80 nanometers and a width of about 375 ± 75 nm and were randomly, yet homogeneously, distributed over the whole film surface, with their margins reminding of fractals. While these structures are too small to be observed forming in detail in the swollen film solutions under the optical microscope, very weak macroscopic changes in the surface homogeneity can be observed for very short times. Such changes usually point toward the material’s structuring [54] but with no possibility of seeing the growth in real time. As a consequence, we suggest that the here-presented gelatin structures nucleated and grew during the C-SVA processing and thus, most probably, exhibit a crystalline nature. Interestingly, the AFM height images further emphasized that all gelatin structures displayed a three-dimensional (3D) appearance (see the inset of Figure 3e), each comprising two fractal-like layered structures (the top structure had an average height of about 16 ± 2 nm) that were stacked on top of each other. Although (bio)polymeric structures reported to grow in the third direction are rather scarce, they were previously shown to form in thin films of polypeptides that were also swollen-rich in solvent vapors [54,55]. The layered gelatin structures were further composed of spherical substructures of an average diameter of around 38 ± 6 nm (Figure 3g). Instead, between the 3D granular structures, the surface of gelatin was covered with much smaller structures of an average lateral dimension of roughly 14 ± 3 nm (Figure 3h). These latter structures were similar to those covering the surface of the reference gelatin films that were obtained by spin casting but were not further exposed to water vapors via C-SVA (Figure 3f,i). In these unexposed films, the surface was smooth, exhibiting no granular structures (Figure 3b,d). While there are many surface morphologies reported in the literature for gelatin, ranging from smooth and/or compact surfaces [57,58] to rough [59], often protrusion and/or aggregate-like structures [60]; from sponge or coral-like features [61] to nanoparticles [62]; and from irregular cavities with interconnections [63] to other (fiber-based) networks [64], to the best of our knowledge, until now, no evidence has been found for the existence of granular 3D crystalline structures such as those reported in this work.

**Figure 4:**
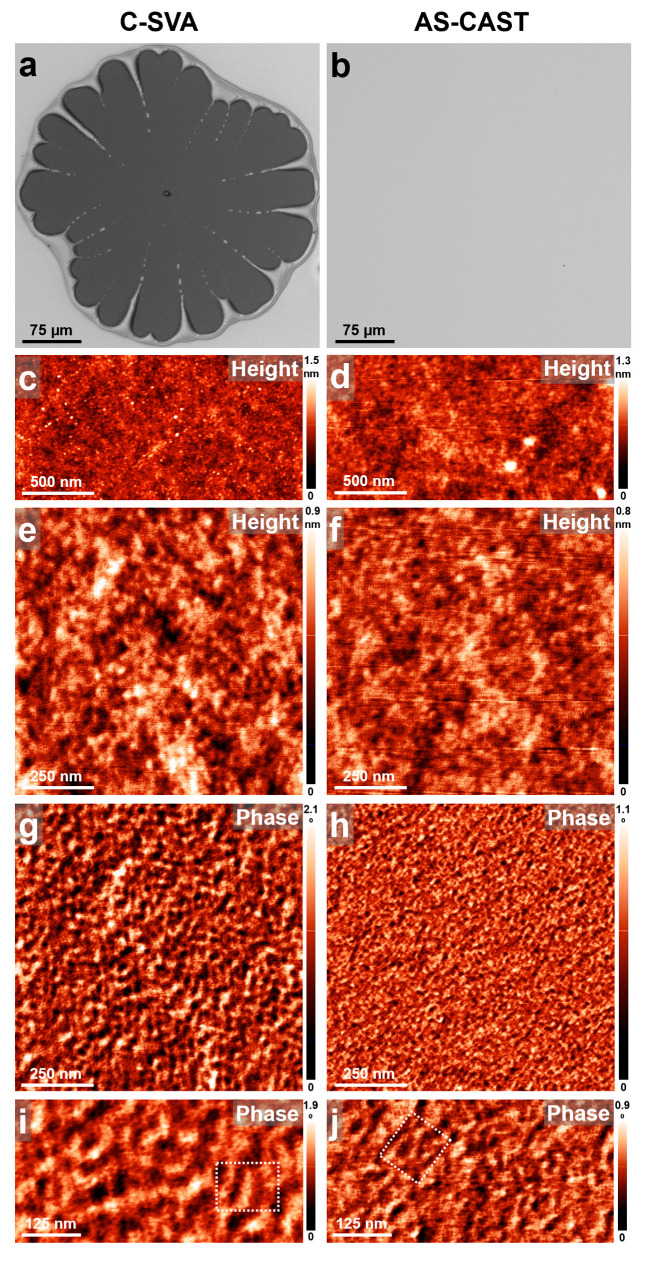
(**a**–**j**) Optical (**a**,**b**) and AFM height (**c**–**f**) and phase (**g**–**j**) images showing the surface morphology of a thin film of dextrin before (**right side**) and after (**left side**) being exposed to ultrapure water vapors via C-SVA. Note that the substructures forming inside the dewetted pattern in (**a**) had an average lateral dimension much larger than the substructures observed on the unprocessed dextrin film in (**b**). The dotted shapes in (**i**,**j**) are for guiding the eye only.

Dextrin was another biopolymer exposed to ultrapure water solvents with the help of the C-SVA method. The optical micrographs depicted in Figure 4a,b, acquired for a dextrin film before and after its processing, clearly show that the C-SVA processing induced the reorganization of dextrin chains. This happened within film areas that experienced dewetting. This latter phenomenon was present because of the rather unstable nature of the dextrin film under the C-SVA processing conditions. Figure 4a emphasizes the resulting morphology of a typical dewetting pattern, exhibiting an average diameter of about several hundreds of micrometers. Obviously, no such patterns were observed on the unexposed dextrin film, suggesting that the latter became unstable upon C-SVA processing (Figure 4b). Nonetheless, when the exposed and unexposed morphologies were further compared using the AFM technique, one could observe that while both morphologies were rather smooth and featureless at the micrometer scale (Figure 4c,d), they exhibited visible differences at a lower length scale (Figure 4e–j). As it was inferred from the high-magnification AFM phase micrographs by analyzing many cross-sectional profiles, the substructures forming inside the dewetted pattern (three such substructures are indicated by the dotted rectangular shape in Figure 4i) had an average lateral dimension of about 41 ± 5 nm. In comparison, the lateral dimension of substructures observed on the unprocessed dextrin film (three such substructures are indicated by the dotted rectangular shape in Figure 4j) was only around 25 ± 5 nm. This observation could be possibly explained by assuming that dextrin chains adopted, upon the C-SVA processing followed by dewetting, a more extended conformation, i.e., a conformation of a lower degree of chain folding. Finally, it is worth mentioning that various yet different microstructures of dextrin, corresponding preponderantly to film configurations, were previously reported in the literature including powder-based granular [65] and egg-like [66] resembling morphologies, smooth featureless surfaces [67,68], homogeneous and/or non-porous [34], and spherical/branched [69] structures.

**Figure 5:**
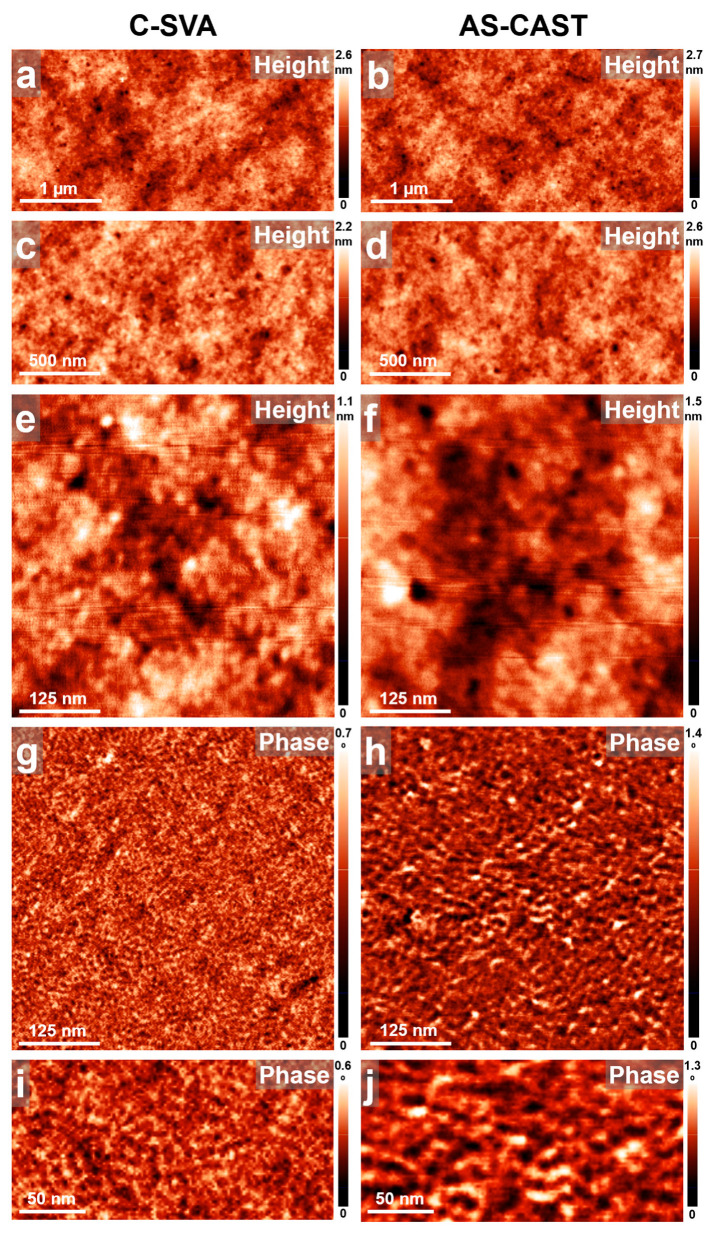
(**a**–**j**) AFM height (**a**–**f**) and phase (**g**–**j**) micrographs illustrating the surface morphology of a thin film of glycogen before (**right side**) and after (**left side**) being exposed to ultrapure water vapors via the C-SVA method. The surface morphology of both C-SVA processed and unprocessed reference films comprised spherical objects of an average diameter of ~9.4 nm and ~13.5 nm, respectively. Note that artifacts such as some noisy lines in (**e**,**f**) cannot be avoided during the AFM measurements conducted while using weak tapping forces (i.e., a very soft tapping mode regime) and are rather normal, especially when acquiring high magnification AFM images of a rather small surface area.

Opposite to gelatin, overall, glycogen molecules generated no large crystalline structures (Figure 5). The surface of the glycogen reference film was pretty smooth and featureless, similar to that of the glycogen film exposed to water vapors using the C-SVA method (Figure 4a–h). Both surfaces were compact, with only a few tiny pores formed randomly, and exhibited a surface roughness at the nanometer level. Nonetheless, when analyzing the AFM phase images of higher magnification in more detail, it was observed that the glycogen molecules may have possibly adopted a slightly different chain conformation upon C-SVA processing in water vapors, as compared with the glycogen chains that generated the reference film. More precisely, while the glycogen structures formed at the molecular level displayed a rather lamellar appearance in both cases, their average lateral dimension (e.g., width) was smaller within the film that was processed via the C-SVA (about 9.4 ± 1 nm), as compared with the average dimension of structures formed within the unexposed reference film (~13.5 ± 2 nm in width; see Figure 5i,j). Interestingly, in both films, the observed lamellae seemed to comprise spherical objects of an average diameter of about 9.4 ± 1 nm and 13.5 ± 2 nm, respectively. Thus, we tentatively concluded that the observed glycogen structures were a result of the self-assembly process that packed glycogen chains into small nanoparticles and then further arranged the latter into curved, lamellar rows, as no evidence for crystallization was found using the optical microscopy and AFM techniques. Our experimental observations seem to be in good agreement with the previous theoretical and experimental findings that reported the tendency of glycogen molecules to form nanoparticles [19,70,71]. Such nanoparticles are generally a result of randomly joined glycogen branches (of a typical dimension of about 2 nm) that form so-called *β*-particles (typically exhibiting a size of ~20 nm) at a larger scale, which in turn can join again on an even larger scale to form ~100 nm sized *α*-particles [19,72,73,74]. Judging by the size of our glycogen nanoparticles, although sensitively smaller, they would still fit within the case of typical *β*-particles (most probably displaying less branching after the processing in ultrapure water vapors via the C-SVA; compare the particle diameters of 9.4 ± 1 nm and 13.5 ± 2 nm). These latter *β*-particles most probably failed to further form larger *α*-particles and instead preferred to line up into curved lamellar structures, as observable in Figure 5i,j.

**Figure 6:**
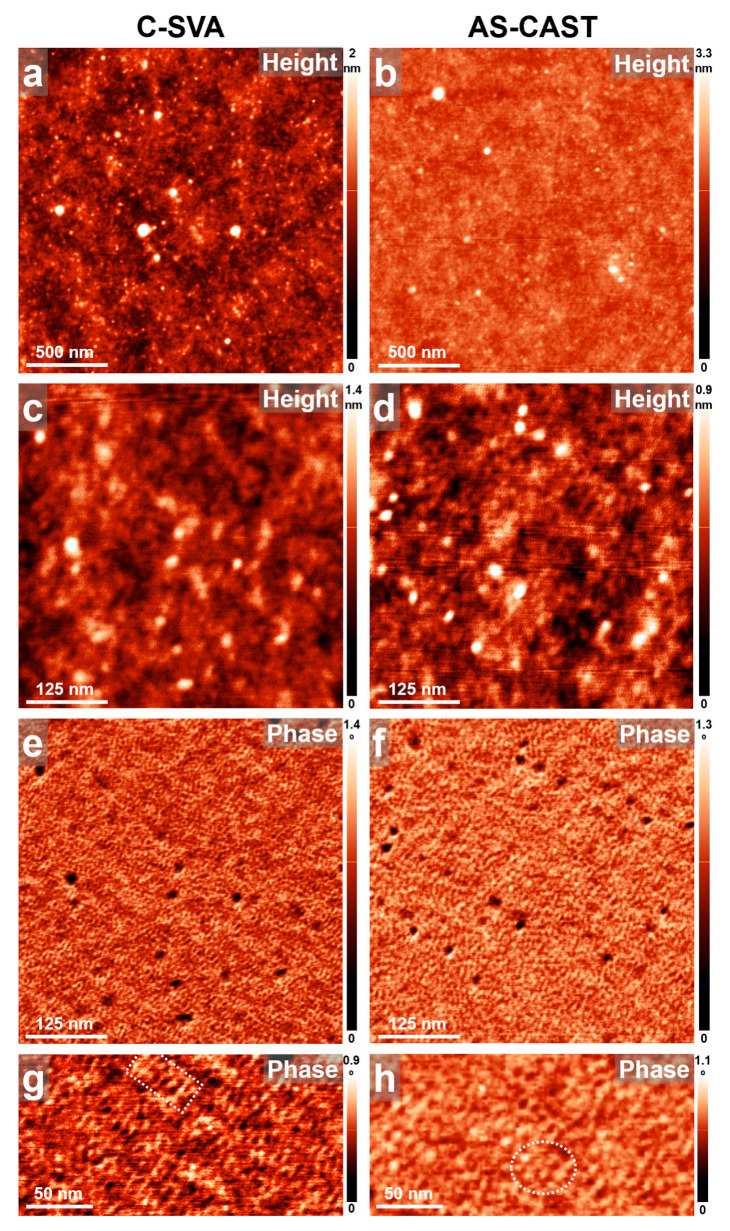
(**a**–**h**) AFM height (**a**–**d**) and phase (**e**–**h**) micrographs depicting the surface morphology of a thin film of chitosan before (**right side**) and after (**left side**) being exposed to acetic acid vapors via the C-SVA method. The dotted shapes in (**g**,**h**) are for guiding the eye only. Note that the surface morphology of chitosan changed upon C-SVA processing in acetic acid vapors from random ~10 nm large spherical structures to smaller spherical structures often assembled into rather straight lamellar rows. Also note that artifacts such as some noisy lines in (**c**,**d**) cannot be avoided during the AFM measurements conducted while using weak tapping forces (i.e., a very soft tapping mode regime) and are rather normal, especially when acquiring high magnification AFM images of a rather small surface area.

Chitosan is another biopolymer known to form nanoparticles [75,76,77], as well as mono- and multi-chamber vesicles [78]. Because of its extremely broad range of applications stretching from biotechnology to pharmaceutics and medicines, membranes and water treatment, cosmetics, foods [79,80], etc., chitosan is a well-studied biopolymer. Therefore, there is a lot of interest in altering and manipulating its corresponding film microstructure. According to various scientific reports, the latter is generally represented by a homogenous smooth and featureless surface [81,82,83,84], often exhibiting granular [85] or spherical [86,87] structures. Nonetheless, the chitosan microstructure in thin films was shown to be strongly dependent also on the film casting conditions and the type of utilized substrate. As a result, chitosan can form uniform network structures composed of fine chains, spherical nanoparticles, dendritic structures, branched chains, or other dense networks comprising small particles [88]. Comparing our results obtained on chitosan films with those reported in the literature, we can indeed confirm that chitosan molecules tended to form spherical structures that covered the whole film surface right after the spin casting process was over (Figure 6, right side). Moreover, the film surface appeared smooth and featureless in lower magnification AFM height images. The average diameter of spherical structures, better visible in Figure 6f,h (and emphasized in the ellipsoidal dotted shape in Figure 6h), was determined to be around 10 ± 1 nm. Interestingly, while the film surface remained smooth and featureless also upon processing the chitosan film in acetic acid vapors via the C-SVA method (see the low-magnification AFM height and phase images in Figure 6a,c,e), a closer view with the AFM technique revealed that the spherical chitosan structures further assembled into more or less straight lamellar rows (several such rows are clearly delimited within the rectangular dotted shape in Figure 6h) of an average width of only 7 ± 1 nm, just like it was previously observed in the case of glycogen. Thus, these experimental observations further demonstrated that exposing thin films of chitosan to ultrapure water vapors induced an additional level of ordering.

**Figure 7:**
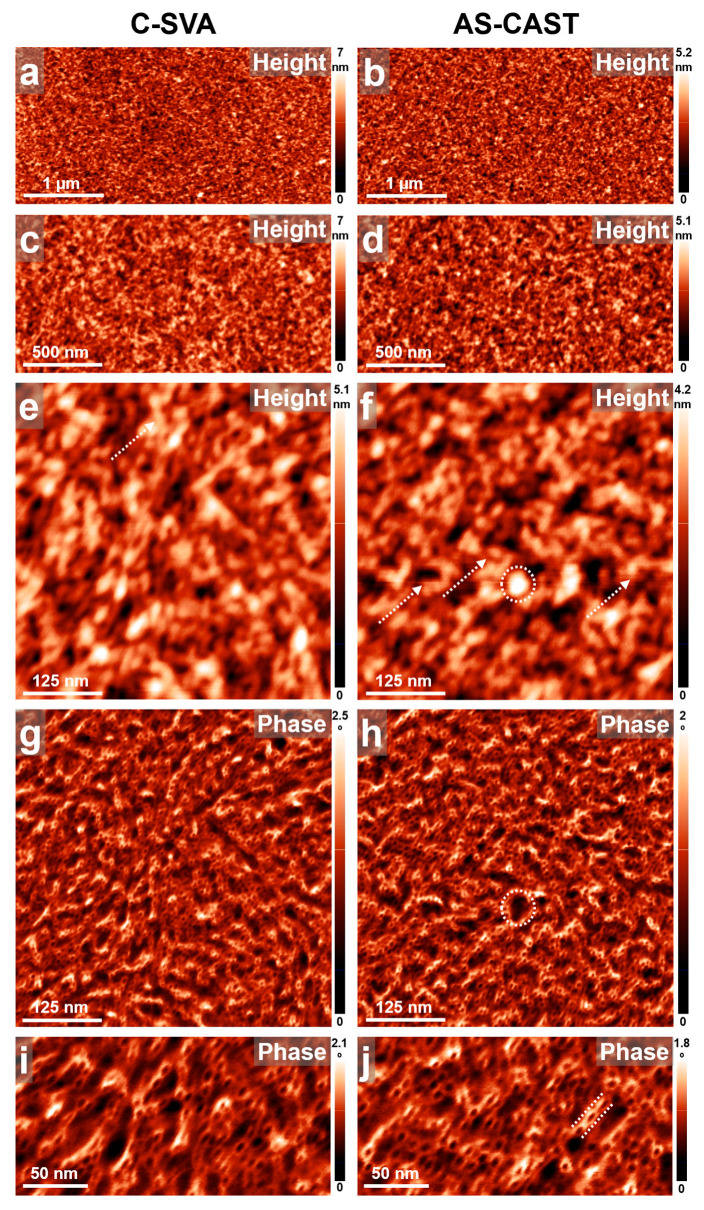
(**a**–**j**) AFM height (**a**–**f**) and phase (**g**–**j**) micrographs depicting the surface morphology of a thin film of phytagel before (right side) and after (left side) being exposed to ultrapure water vapors via the C-SVA method. In (**i**,**j**), interhelical associations among bundles of double helices can be observed. Note that while a few noisy artifact lines in (**e**,**f**), indicated by the white arrow, could not be avoided while acquiring the AFM height images, these lines are rather normal when performing AFM measurements in a very soft tapping mode regime (employing weak tapping forces) over a rather small area. The dotted circular shapes in (**f**,**h**), as well as the dotted arrows in (**e**,**f**) and lines in (**j**), are for eye guidance only.

Furthermore, we also conducted extended AFM studies on thin films of phytagel (also known as gellan gum) molecule. The results are presented in Figure 7. While we found no visible influence of C-SVA processing on the film microstructure of this particular biomolecule (the surface of the films was smooth and compact, exhibiting no particular structures at the micrometer level; compare the left and right side of Figure 7), we decided nonetheless to present the corresponding AFM images, as they depict the phytagel system in unprecedented detail and reveal that phytagel molecules self-assemble right after the spin casting process into narrow helical structures exhibiting an average width of ~11 ± 2 nm and an average height of few nanometers (see one of such structures emphasized in Figure 7j in between the two dotted white lines). Again, no differences were visible when comparing a C-SVA-processed phytagel film with its unprocessed analog (compare Figure 7i with Figure 7j). To the best of our knowledge, these helix-resembling structures of phytagel systems in the thin film configuration were not visualized in such detail in the literature until now. While a crystal structure of gellan was deciphered by means of X-ray diffraction and shown to comprise three-fold helical chains assembled in parallel with each other in an intertwined duplex, with each chain being translated half a pitch with respect to the other [89], only low-magnification AFM images depicting phytagel fibrils and fibrillar networks were reported [90,91,92,93]. Such phytagel fibrils were suggested to form through an initial arrangement of single chain phytagel molecules into ordered double helices which, in turn, merged and generated interhelical associations [90]. Obviously, phytagel molecules could retain their double-helical structure too, only to form well-defined filaments [94] (note that other reported structures of phytagel included only 3D cross-linked networks [95], interconnected gel grains [96], or hydrogel beads [36]). Nonetheless, the helical nature of such fibrils or filaments has not been visible in AFM micrographs so far. Note that the AFM images were acquired using a high set point that maintained a consistently soft tapping regime (i.e., weak tapping forces were employed) that circumvented any deformation of the surface, simultaneously avoided the attachments of amorphous phytagel molecules to the tip, and also allowed us to obtain a good phase contrast with clear details of the molecular arrangements at the nanoscale. Also further note that, while some higher structures appeared darker (e.g., softer) in the phase images (see the dotted circular shapes in Figure 7f,h), these structures were featureless (i.e., no helical structures were present), and thus most probably comprised soft amorphous/coiled-like molecular arrangements. Moreover, taking into account all these data and considering that fibrous structures of cross-sectional heights around 1 nm are considered to consist of bundles of laterally aggregated double helices [92], we tentatively suggest that the 11 ± 2 nm wide and few nanometers tall helix-resembling structures of phytagel shown in Figure 7 represent interhelical associations of double-helix bundles.

**Figure 8:**
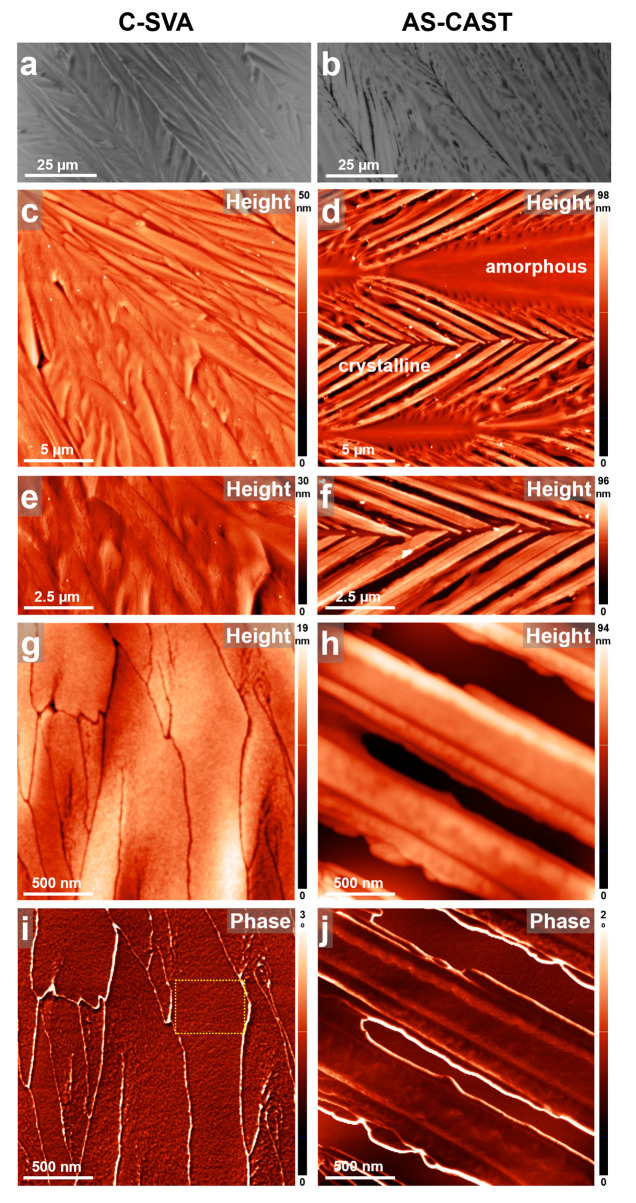
(**a**–**j**) Optical (**a**,**b**) and AFM height (**c**,**h**) and phase (**i**,**j**) micrographs illustrating the surface morphology of a thin film of dopamine hydrochloride before (**right side**) and after (**left side**) being exposed to ultrapure water vapors via the C-SVA method. More compact columnar dendritic structures, with almost no amorphous material, were obtained upon processing dopamine via the C-SVA method. The dotted shape in (**i**) indicates the area that was further magnified in the next Figure 9.

Dopamine hydrochloride was the last biological monomeric system that we processed in ultrapure water vapors using the C-SVA method and further investigated for surface microstructure with the AFM technique (see the obtained results summarized in Figure 8). Right after the spin-casting of thin dopamine hydrochloride films, under the optical microscope, we observed that many columnar dendritic structures self-assembled all over the film surface (Figure 8b). These dendritic columns were further separated by clearly visible empty regions. When looking more closely with the AFM technique, the obtained reference dopamine film displayed many amorphous regions surrounded by crystalline columnar branches of, more rigid nature (Figure 8d,f). Most probably, the separation of columnar branches by the large empty regions (better visible in Figure 8h) was a result of the competition between the neighboring self-assembling dendritic branches for the available molecules in the surrounding reservoir. Moreover, the majority of dendritic columnar branches exhibited highly crystalline and, thus, very rigid margins, as can be deduced from the AFM phase image presented in Figure 8j. Here, these margins displayed a very bright color. Unfortunately, because of (i) this intense contrast, (ii) the large variations in height across lateral dimensions smaller than 500 nm displayed on the surface of this reference film, and (iii) the omnipresence of some amorphous/sticky molecules on top of the columnar branches (amorphous molecules were often sticking to the AFM tip), it was not possible to image such branches clearly at a higher AFM magnification than that presented in Figure 8h,j.

The here observed columnar dendritic structures are not a novel element for dopamine. It is well known in the literature that (columnar) dendrites, spoke-like, flower-like, or various fractal-like structures can be self-assembled from polydopamine [97]. Note here that dopamine, in certain pH and temperature conditions or in the presence (or content) of other assisting materials (chemical groups), is well-known to self-polymerize and form so-called polydopamine [97,98,99]. Considering that we used ultrapure water solvent pushed by nitrogen gas in our experiments, with a pH in this case around 7 [100], it was thus expected that our dopamine hydrochloride starting material would self-polymerize into polydopamine and, thus, further self-assemble upon the relatively short deposition time during spin casting (as it can be observed on the right side of Figure 8). Therefore, the columnar dendric structures presented in Figure 8 are, for instance, similar to the structures reported not long ago by Zhang and co-workers [97].

**Figure 9:**
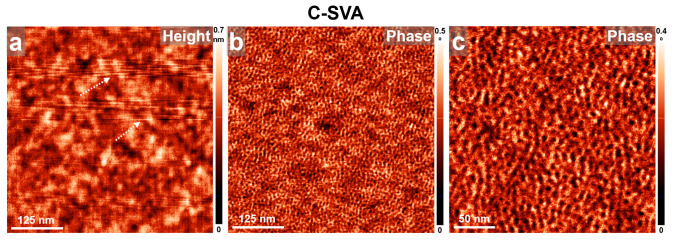
(**a**,**b**) High-detail AFM height (**a**) and phase (**b**) micrographs illustrating the lamellar-resembling surface morphology self-assembled in a thin film of dopamine hydrochloride that was exposed to ultrapure water vapors via the C-SVA method. (**c**) AFM phase image zooming in a region of (**b**). Note that some noisy artifact lines in (**a**), indicated by dotted white arrows, could not be avoided while acquiring the AFM height image in a very soft tapping mode regime (and employing weak tapping forces) over a rather small, yet very uniform area. These artifacts are much less prominent in the corresponding phase image and do not impact the obtained AFM results.

The novelty of studying such polydopamine films arose when we went one step forward and exposed the latter to ultrapure water vapors utilizing the C-SVA method. As a result, more compact columnar dendritic structures were obtained (see the optical micrograph presented in Figure 8a). Here, there were no more large empty regions present in between the columnar branches, a sign that polydopamine molecules of increased mobility could freely move in the rich-swollen “film solution” and timely arrive at the “structure assembling site”. This observation was further supported by the AFM height micrographs shown in Figure 8c,e. Nonetheless, very narrow regions separating the self-assembled compact branches appeared to be visible only in high-magnification AFM height and phase images (Figure 8g,i). Furthermore, because of the more compact structure of the branches, it was possible to identify at least 1 × 1 µm^2^ large areas that were very smooth and not covered with any detectable (amorphous) sticky molecules (see one of such areas delimited by the dotted square shape in Figure 8i). Consequently, we were able to acquire high-magnification AFM images and provide new details on the polydopamine microstructure (Figure 9). As we can better observe in Figure 9c, the polydopamine system self-assembled into curved lamellar-resembling structures of an average lateral dimension of ~8 ± 1.5 nm. This value was inferred from the analysis of several cross-sectional profiles taken along various directions in Figure 9c, and it was significantly smaller than the value of granular features (displaying a diameter of 25 ± 5 nm [99], similar [101], or larger [102,103]) commonly reported in the literature. To the best of our knowledge, no lamellar-resembling structures of polydopamine were reported before and depicted at this magnified scale.

## 4. Conclusions

In this work, we exposed thin films of various biopolymers, such as gelatin, dextrin, glycogen, chitosan, and phytagel, to solvent vapors until a rich film-swelling regime was attained. In this regime, biopolymer molecules displayed increased mobility and were prone to crystallize or self-assemble into various structures composed of more ordered chain conformations. As revealed by the AFM studies, the gelatin biopolymer preferred to crystallize into granular compact structures. The other studied biopolymers self-assembled either into (curved) lamellar-resembling structures composed of spherical nanoparticles (in the cases of glycogen and chitosan) or into more complex helix-resembling morphologies (in the case of phytagel). The AFM studies presented here are important because they either report novel surface structures of biopolymers (e.g., crystals of gelatin) or depict, in unprecedented detail, surface morphologies composed of interhelical associations of bundles of double phytagel helices. Moreover, while the efficiency of the rich swelling of thin (bio)polymeric films in solvent vapors was demonstrated some time ago on various polypeptide homopolymers, diblock, and hetero-arm star block copolymers (see ref. [54,55]), the optimization of this processing method to C-SVA gave us the ability to alter and control the morphology of other diblock and triblock copolymers (see ref. [56]) with the help of both self-assembly and crystallization processes. In all these studies, one has to be aware that while the process of film swelling is the same no matter which polymeric system is processed (e.g., condensation of warmer solvent vapors on the colder film surface), the physical parameters like the film temperature when the condensation starts or the amount of condensed solvent vapors, critically depend on the type of utilized solvent (and thus, on its volatility), on its temperature or the amount of vapors introduced in the sample chamber. Therefore, some limitations of the here-proposed C-SVA tool are related to the use of solvents with very low volatility that would require condensation to happen at temperatures lower than 10–12 °C or of bad solvents unable to fully dissolve the polymer film, thus failing to generate a homogeneous film solution. This work further completes the spectrum of the applicability of the C-SVA method on certain biopolymers, as well as simple monomer materials (e.g., dopamine hydrochloride), a potential that could be exploited in the future when designing and developing novel biopolymer-based applications, especially when targeting applications highly sensitive to the molecular arrangements such as drug delivery or sensing.

It is also worth noting that control over the microstructure of biopolymers is crucial in biomimetics as it could allow for the replication of natural structures found in living organisms, leading to the development of innovative materials with unique properties. For instance, biopolymers based on gelatin, chitosan, or cellulose are desired to be structured at both the microscale and nanoscale levels in order to create biomimetic cellular architectures that could further enhance the mechanical properties of biopolymer films, increasing their stiffness and strength or to alter their interactions with other components (water interactions, ionic concentration, etc.). Moreover, in tissue engineering, biopolymer scaffolds comprising structured nanofibrillar objects could be used to mimic natural extracellular matrices, thus providing an ideal biomimetic environment for the growth of cells and eventually for tissue regeneration.

## Figures and Tables

**Table 1:** Summary of various physical and chemical parameters characterizing both the starting biopolymer systems and their corresponding thin films.

Biopolymer	Molecular Weight (g/mol)	Solvent Type	Solubility (mg/mL)	Film Thickness (nm)	Film Stability during C-SVA
Dextrin	-	water	50	111 ± 7	rather unstable
Glycogen	-	water	-	116 ± 8	stable
Phytagel	1,000,000	water	10	93 ± 5	stable
Gelatin	-	water	50	89 ± 5	stable
Chitosan	190,000–310,000	acetic acid	-	86 ± 5	stable
Dopamine	189.64	water	100	97 ± 6	stable

## Data Availability

The data are contained within this article; additional data are available on request from the corresponding author.
